# Mouse Umbilical Cord Mesenchymal Stem Cell Paracrine Alleviates Renal Fibrosis in Diabetic Nephropathy by Reducing Myofibroblast Transdifferentiation and Cell Proliferation and Upregulating MMPs in Mesangial Cells

**DOI:** 10.1155/2020/3847171

**Published:** 2020-05-02

**Authors:** Hongde Li, Pengfei Rong, Xiaoqian Ma, Wei Nie, Yan Chen, Juan Zhang, Qiong Dong, Min Yang, Wei Wang

**Affiliations:** ^1^Cell Transplantation and Gene Therapy Institute, The Third Xiangya Hospital of Central South University, Changsha, Hunan, China; ^2^Department of Radiology, The Third Xiangya Hospital of Central South University, Changsha, Hunan, China; ^3^Postdoctoral Research Station of Special Medicine, The Third Xiangya Hospital of Central South University, Changsha, Hunan, China; ^4^Department of Pathology, The Third Xiangya Hospital of Central South University, Changsha, Hunan, China; ^5^Engineering and Technology Research Center for Xenotransplantation of Hunan Province, Changsha, China

## Abstract

Transplantation of umbilical cord mesenchymal stem cells (UC-MSCs) is currently considered a novel therapeutic strategy for diabetic nephropathy (DN). However, the mechanisms by which UC-MSCs ameliorate renal fibrosis in DN are not well understood. Herein, we firstly investigated the therapeutic effects of mouse UC-MSC infusion on kidney structural and functional impairment in streptozotocin- (STZ-) induced diabetic mice. We found that the repeated injection with mUC-MSCs alleviates albuminuria, glomerulus injury, and fibrosis in DN mouse models. Next, mesangial cells were exposed to 5.6 mM glucose, 30 mM glucose, or mUC-MSC-conditioned medium, and then we performed western blotting, immunofluorescence, wound healing assay, and cell proliferation assay to measure extracellular matrix (ECM) proteins and matrix metalloproteinases (MMPs), myofibroblast transdifferentiation (MFT), and cell proliferation. We demonstrated that mUC-MSC paracrine decreased the deposition of fibronectin and collagen I by inhibiting TGF-*β*1-triggered MFT and cell proliferation mediated by PI3K/Akt and MAPK signaling pathways, and elevating the levels of MMP2 and MMP9. Importantly, we provided evidence that the antifibrosis role of mUC-MSC paracrine in DN might be determined by exosomes shed by MSCs. Together, these findings reveal the mechanisms underlying the therapeutic effects of UC-MSCs on renal fibrosis in DN and provide the evidence for DN cell-free therapy based on UC-MSCs in the future.

## 1. Introduction

Diabetic nephropathy (DN), also called diabetic glomerular sclerosis, is a serious microvascular complication caused by diabetic mellitus (DM) and is the leading reason for end-stage renal disease (ESRD) [1, 2]. DN is characterized by specific renal morphological and functional alterations. Among them, the early stage of DN features glomerular hyperfiltration, hypertrophy, microalbuminuria, basement membrane thickening, and mesangial expansion. The features of advanced DN are a progressive decline in glomerular filtration rate (GFR), macroalbuminuria, decreasing creatinine clearance, and glomerular and tubular-interstitial fibrosis.

Renal fibrosis is a common outcome of almost all chronic kidney diseases, eventually leading to irreversible kidney damage. The accumulation of mesangial matrix is an important pathological basis for glomerular fibrosis. Numerous studies have shown that TGF-*β*1, the key profibrotic factor, can promote the synthesis of extracellular matrix (ECM), thereby accelerating the process of renal fibrosis [3–5]. Emerging evidence indicates that renal fibrosis is associated with profibrotic alterations in intrinsic kidney cells through epithelial-mesenchymal transition (EMT), endothelial-mesenchymal transition (EndoMT), and myofibroblast transdifferentiation (MFT) [6, 7]. TGF-*β*1 mainly triggers the transdifferentiation of the intrinsic renal cells through downstream Smad2/3-dependent signaling pathway [8, 9]. In addition, TGF-*β*1 downstream Smad2/3-independent signaling pathways, including MAPKs [10], PI3K/Akt [11], RhoA [12], and Wnt/*β*-catenin [13], contribute to renal fibrosis. And also, matrix metalloproteinases (MMPs), known for their function in the degradation of ECM proteins [14], have been identified as the potential therapeutic targets for kidney fibrosis.

At present, there is no satisfactory method for the treatment of DN. It is noticeable, however, that the reparative and regenerative therapeutic strategies for treating DN have drawn more attention. There is a growing body of evidence that mesenchymal stem cells (MSCs) prevent the progression of DN [15] and overt albuminuria [16, 17]. Moreover, MSCs derived from the umbilical cord (UC-MSCs) exhibit lower immunogenicity, higher proliferation potential and differentiation capability, and faster self-renewal than other tissue-derived MSCs, which make them a broad application prospect [18]. An animal study has shown that human UC-MSCs (hUC-MSCs) improved glomerular hypertrophy, basement membrane thickening, and urinary protein and creatinine clearance in DN rats [19]. In vitro, coculturing with hUC-MSCs ameliorates podocyte apoptosis induced by high glucose, probably by secreting the soluble hepatocyte growth factor (HGF) [20]. The infusion of hUC-MSCs has been proven safe and effective to treat DN in only one clinical trial. And the therapeutic effect is better than the conventional method in improving renal function [21]. Recently, Liu et al. have demonstrated that conditioned medium (CM) from hUC-MSCs plays an important role in the attenuation of renal fibrosis by reducing inflammation and EMT [22]. Because only a little research has focused on the effects of UC-MSCs on renal fibrosis in DN, the underlying mechanisms are still not well understood.

Here, we demonstrate that repeated administration of mUC-MSCs attenuates the progression of DN by improving glomerular hypertrophy, base membrane thickening, podocyte process effacement, and fibrotic abnormality in STZ-induced diabetic mice. mUC-MSC paracrine can alleviate renal fibrosis in the DN cell model via inhibition of MFT caused by TGF-*β*1, blocking mesangial cell proliferation induced by PI3K/Akt and MAPK signaling pathways, and elevating the levels of MMP2 and MMP9. It is worth noting that we are the first to provide the proof that the antifibrotic effect of mUC-MSC paracrine might be mainly due to exosomes, which adds to our understanding of the role of UC-MSC paracrine in alleviating renal fibrosis in DN and also provides the evidence for the future cell-free therapeutic strategy based on UC-MSCs for DN.

## 2. Materials and Methods

### 2.1. Cell Culture and Administration

The mouse umbilical cord mesenchymal stem cell line mUC-MSC (BNCC340370) and mouse mesangial cell line SV40-MES-13 (ATCC^CRL-1927^) were purchased from BeNa Culture Collection (BeNa Chuanglian Biotechnology Research Institute, Beijing, China) and American Type Culture Collection (Manassas, VA 20108, USA), respectively. Both cell lines were cultured in DMEM medium (HyClone, Thermo Fisher Scientific, Logan, Utah, USA) supplemented with 10% FBS (EVERY GREEN, Zhejiang Tianhang Biotechnology Co. Ltd., Zhejiang, China), 100 U/mL penicillin, and 100 *μ*g/mL streptomycin (Beijing Solarbio Science & Technology Co., Ltd., Beijing, China) in a humidified atmosphere of 5% CO_2_ at 37°C.

Some of the SV40-MES-13 cells were placed in 6-well plates and cultured with normal glucose (NG, 5.6 mM) or high glucose (HG, 30 mM) for 24 hours. Meanwhile, the other SV40-MES-13 cells were seeded in the transwell chamber and then placed on a 6-well plate that contained mUC-MSCs. After coculturing for 24 hours, the SV40-MES-13 cells were harvested for further analyses.

### 2.2. Differentiation of mUC-MSCs

To induce adipogenic differentiation, confluent cells were cultured in a UC-MSC-induced adipogenic differentiation medium (Cyagen Biosciences, China). According to the instructions and procedures of a UC-MSC-induced adipogenic differentiation medium kit, after about 20 days, cell differentiation into lipid-laden adipocytes was confirmed by Oil Red O staining (Cyagen Biosciences, China). For osteogenic differentiation, adherent cells were grown at 2 × 10^4^ cells/cm^2^ in a UC-MSC-induced osteogenic differentiation medium (Cyagen Biosciences, China). According to the instructions and procedures of a UC-MSC-induced osteogenic differentiation medium kit, after 17~31 days of culture, calcium deposits were detected by Alizarin Red staining (Cyagen Biosciences, China). To induce chondrogenic differentiation, 3‐4 × 10^5^ cells in a 15 mL centrifuge tube were centrifuged at 150 g for 5 min and then incubated for 24~48 hours in 0.5 mL of culture medium to achieve conditions for micromass formation. According to the instructions and procedures of a UC-MSC-induced chondrogenic differentiation medium kit, the micromass was cultured in a UC-MSC-induced chondrogenic differentiation medium (Cyagen Biosciences, China) for 21~28 days, and chondrogenic differentiation was assessed by Alcian Blue staining (Cyagen Biosciences, China).

### 2.3. Preparation of Conditioned Medium from mUC-MSCs

The mUC-MSCs (passage 4) were seeded into 10 cm culture dishes. When the confluence was reached, the medium was changed to a serum-free medium. After 24 h, the supernatants were collected to remove cell debris by a centrifuge and a 0.45 *μ*m filter. Then, the medium without cell debris was supplemented with FBS, 100 U/mL penicillin, 100 *μ*g/mL streptomycin, and additional glucose to reach 30 mM, thereby producing an mUC-MSC-conditioned medium (mUC-MSC-CM).

### 2.4. Animal Models of Diabetic Nephropathy and Administration of mUC-MSCs

Male BALB/C mice, weighing 20~25 g at 8 weeks of age, were purchased from Hunan SJA Laboratory Animal Co. Ltd. (Hunan, China). The mice were kept in cages, given 12-hour light/dark cycles, and given free access to a standard diet and water throughout the experiment. All animal studies were performed in accordance with the laboratory animal care and use guidelines of Central South University. This study was approved by the Institutional Animal Care and Use Committee of Central South University. The mice were randomly divided into three groups: (A) The normal group (*n* = 6) is composed of mice intraperitoneally injected with 200 *μ*L of sodium citrate buffer (0.01 M, pH 4.2~4.4). (B) The diabetic mellitus (DM) group (*n* = 8) is composed of mice intraperitoneally injected with 150 mg/kg streptozotocin (STZ, Sigma-Aldrich, St. Louis, MO) dissolved in 200 *μ*L of sodium citrate buffer once. Two weeks after STZ injection, the mice with glucose concentration greater than or equal to 16.7 mM were recruited in this study. The recruited diabetic mice were injected with phosphate buffer solution (PBS) via the tail vein 4 weeks after STZ injection. (C) The DM+MSC group (*n* = 8) is composed of recruited diabetic mice injected with mUC-MSCs via the tail vein. All the animals in the three groups were sacrificed after 8 weeks of injection with mUC-MSCs, and then urine and kidney tissue samples were taken for further analysis.

The mice of the MSC group were injected with 200 *μ*L of mUC-MSCs (1.0 × 10^4^ MSCs/g body weight per animal suspended in PBS) via the tail vein weekly for 4 weeks. The mice of the diabetic group were instead injected with the same volume of PBS via the tail vein.

### 2.5. Cell Proliferation Assays

The mesangial cells were seeded into 96-well plates at 2 × 10^3^/well in triplicate. The cell proliferation was determined by using the CellTiter 96® AQueous One Solution Cell Proliferation Assay (MTS) (Promega, Madison, WI, USA) according to the manufacturer's protocol and each group was repeated four times. MTS solution was added to each well at 0 h, 12 h, 24 h, 36 h, and 48 h and incubated for 2 h. The absorbance of mesangial cells was measured at 490 nm using a microplate reader (BioTek Epoch ELISA, USA).

### 2.6. Flow Cytometry Analysis

The mouse UC-MSC cells (passage 4) were collected and washed with PBS to achieve a single cell suspension. Then, the cells were incubated with the following monoclonal antibodies against surface antigens (BD Biosciences, USA): CD73-FITC, CD90-FITC, CD105-PE, CD11b-PE, CD34-PE, and CD45-PE. Finally, the incubated cells were washed and resuspended in 0.5 mL of PBS for analysis using a FACSCalibur flow cytometer (Beckman Coulter Life Sciences, USA).

### 2.7. Optical Microscope Observations of Renal Tissues

Kidney was fixed in 4% paraformaldehyde and embedded in paraffin. Paraffin-embedded renal tissues were cut into thin sections (3 *μ*m), deparaffinized, and rehydrated. For morphometric analysis of the glomeruli, the renal tissue sections were stained with hematoxylin-eosin (H&E), periodic acid schiff (PAS), and Masson trichrome (MT), respectively, and then observed with an optical microscope. The whole-glomerular tuft volume was measured according to the reported method [23]. In each analysis, 50 sections were examined from 3 animals in each examination.

### 2.8. Transmission Electron Microscopic Examination

The renal cortical tissue specimens were sliced into 1 × 1 × 3 mm^3^ in size and double-fixed in 2.5% glutaraldehyde solution with Millonig's Phosphate Buffer (pH = 7.3). Dehydration of the samples were carried out at room temperature in a graded series of 50%, 70%, and 90% acetone at 10 min intervals for each step followed by 100% acetone twice at 15 min intervals. Sample resin soaking and embedding process was carried out on the specimens in a 1 : 1 mix of acetone : resin for 12 hours and in 100% resin to polymerize overnight at 37°C. Sample resin solidifying process was the carried out on the specimens in 100% resin to polymerize overnight at 37°C and then for 12 hours at 60°C. 50-100 nm ultrathin sections of specimens were made with an ultramicrotome and a diamond knife. After 3% uranyl acetate and lead nitrate double staining, the specimens were examined and photographed with an electron microscope.

### 2.9. Microalbuminuria Levels Detected by Enzyme-Linked Immunosorbent Assay (ELISA)

After injection with mUC-MSCs, 24 h urine samples of mice in each group were collected for detecting the levels of microalbuminuria at 0 wk, 1 wk, 2 wk, 4 wk, 6 wk, and 8 wk. The levels of microalbuminuria in urine were determined using a mouse microalbuminuria ELISA kit according to the manufacturer's instructions. The absorbance was measured at 450 nm using a microplate reader (BioTek Epoch ELISA, USA).

### 2.10. Immunofluorescence Staining

The cells were washed with PBS, fixed in 4% paraformaldehyde, and incubated with blocking buffer at room temperature. Next, the cells were incubated with primary antibodies overnight at 4°C, followed by incubation with secondary antibodies for 2 hours at 37°C. The following antibodies and dilutions were used: anti-collagen I antibody (Abcam, USA), anti-fibronectin antibody (Abcam, USA), anti-vimentin antibody (Cell Signaling Technology, USA), anti-E-cadherin antibody (Abcam, USA), and anti-*α*-SMA antibody (Cell Signaling Technology, USA). Nuclei were stained with 4′,6-diamidino-2-phenylindole (DAPI) for 15 min at room temperature. Laser scanning confocal microscope (Leica, Germany) was used to examine the stained cells.

### 2.11. Wound Healing Assay

The mesangial cells were seeded into 6-well plates at 1 × 10^6^/well in triplicate. After having been cultured to confluence, multiple scratches with the same width were made using a 200 *μ*L pipette tip and washed by PBS three times. Then, the mesangial cells were administrated with normal glucose medium, high glucose medium, and mUC-MSC-CM supplemented with 2% for 24 h, respectively, and observed at 0 h, 12 h, and 24 h using an optical microscope.

### 2.12. Western Blotting Analysis

Cell lysates (50 *μ*g) were separated by SDS-PAGE gel and transferred to polyvinylidene difluoride membranes. The membranes were incubated with blocking buffer and each primary antibody overnight at 4°C. After having been washed with phosphate buffer solution with Tween® 20 (PBST), the membrane was incubated with the conjugated secondary antibody at room temperature for 1 hour. Finally, the protein bands were visualized using enhanced chemiluminescence (ECL, Beijing Solarbio Science & Technology Co., Ltd., Beijing, China) reagents. The primary antibodies used here were as follows: anti-TGF-beta 1 antibody (Abcam, USA), anti-collagen I antibody (Abcam, USA), anti-fibronectin antibody (Abcam, USA), anti-Smad2/3 antibody (Cell Signaling Technology, USA), anti-phospho-Smad2/3 antibody (Cell Signaling Technology, USA), anti-vimentin antibody (Cell Signaling Technology, USA), anti-E-cadherin antibody (Abcam, USA), anti-*α*-SMA antibody (Cell Signaling Technology, USA), anti-MMP2 antibody (Abcam, USA), anti-MMP9 antibody (Abcam, USA), anti-PI3K antibody (Cell Signaling Technology, USA), anti-phospho-PI3K antibody (Cell Signaling Technology, USA), anti-Akt antibody (Cell Signaling Technology, USA), anti-phospho-Akt antibody (Cell Signaling Technology, USA), anti-P38 antibody (Santa Cruz Biotechnology, Inc., USA), anti-ERK1/2 (Santa Cruz Biotechnology, Inc., USA), and Phospho-MAPK Family Antibody Sampler Kit (Cell Signaling Technology, USA). The internal standard was *β*-actin (Santa Cruz Biotechnology, Inc., USA).

### 2.13. Statistical Analysis

Analysis of the experimental data was performed using the statistical software GraphPad Prism 8 (GraphPad Software, Inc., La Jolla, USA). Values are expressed as the mean ± SD. Groups were compared using the one-way ANOVA test. Differences were considered statistically significant (*P* < 0.05).

## 3. Results

### 3.1. mUC-MSC Phenotype

**Figure 1:**
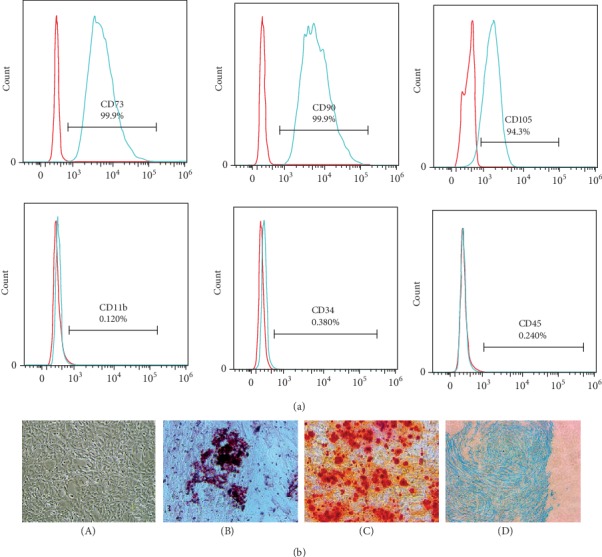
Characteristics of mUC-MSCs. (a) Immunophenotypic characterization of mUC-MSCs (passage 4) was performed by flow cytometry. (b) mUC-MSCs displayed multilineage differentiation potential, differentiating into adipocytes, as indicated by the presence of lipid droplets stained with Oil Red O (magnification × 200); osteocytes, as evidenced by Alizarin Red staining (magnification × 200); and chondrocytes, as shown by the presence of Alcian Blue staining (magnification × 200). (A) Mouse UC-MSCs; (B) Oil Red O stain; (C) Alizarin Red stain; (D) Alcian Blue stain.

As the criterion to identify MSCs, we performed flow cytometry to measure the surface antigen expression in mUC-MSCs. As shown in Figure 1(a), mUC-MSCs were positive for CD73, CD90, and CD105 antigens and negative for CD11b, CD34, and CD45 antigens. When cultured in adipogenic, osteogenic, or chondrogenic medium, mUC-MSCs could exhibit the phenotypic characteristics of an adipocyte, an osteoblast, or a chondrocyte (Figure 1(b)). Taken together, the characterization of mUC-MSCs meets the criteria for defining multipotent MSCs.

### 3.2. Transplantation of mUC-MSCs Improves Renal Function and Injuries to Glomeruli in STZ-Induced Diabetic Mice

**Figure 2:**
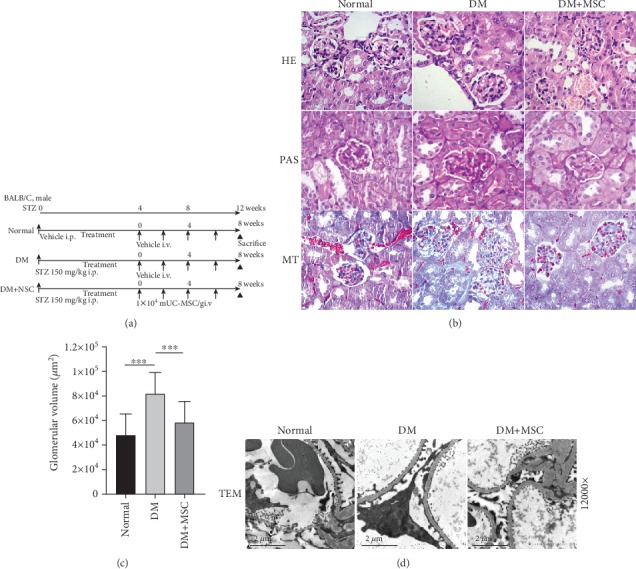
Representative photomicrographs of kidney sections from mice of the different experimental groups, 8 weeks after transplantation of mUC-MSCs. (a) Experimental protocol for mUC-MSC therapies in streptozotocin- (STZ-) induced diabetic mice. (b) Histological findings of the renal cortex in H&E, PAS, and MT staining kidney sections at 8 weeks after the initial administration of mUC-MSCs in STZ-induced diabetic mice. Bar: 200 *μ*m. (c) Quantification of glomerular volume in mice from normal, diabetic, and MSC groups. Data shown are representative of five panels per animal at ×100 magnification. Data are expressed as mean ± SD of 4–6 animals. ^∗∗∗^*P* < 0.001. (d) Ultrastructural TEM analysis of the renal glomerulus in STZ-induced diabetic mice 8 weeks after initial administration of mUC-MSCs. Bar: 2 *μ*m.

The experimental protocol for mUC-MSC therapy in diabetic mice is shown in Figure 2(a). Four weeks after diabetic mellitus (DM) induction, mice presented abnormally high levels of kidney/body weight, blood glucose, and 24-hour urine microalbumin and low level of urine creatinine compared to normal mice (Normal). In this condition, DM mice were randomly assigned into two groups: one group that received the vehicle (DM mice) and another group that received 1 × 10^4^ mUC-MSCs/g weight/week (DM+MSC mice). After 8 weeks of mUC-MSC administration, compared to DM mice, repeated infusion by mUC-MSCs significantly improved abnormal blood glucose, 24-hour urine microalbumin, and urine creatinine levels (Table 1).

We also investigated whether mUC-MSCs were able to improve the abnormal morphological alterations in the renal cortex of DN mouse models. Histological alterations in kidney tissue were evaluated by conventional HE, PAS, and Masson's trichrome staining and by transmission electron microscopy (TEM) observation. Kidneys from DM mice showed glomerular hypertrophy, base membrane thickening, and fibrotic changes compared with kidneys from normal mice. By contrast, repeated injection with mUC-MSCs effectively reduced these abnormal morphological alterations of the kidney in DM+MSC mice (Figure 2(b)). Statistical analysis showed that glomerular volume was significantly augmented in DM mice compared to normal mice, while mUC-MSC transplantation effectively decreased the levels of glomerular volume in DM+MSC mice (*P* < 0.001) (Figure 2(c)). Ultrastructural observation by TEM showed the podocyte foot process effacement and base membrane thickening in DM mice compared to normal mice, and transplantation of mUC-MSCs improved the abnormalities in the glomerulus of DM+MSC mice (Figure 2(d)).

### 3.3. mUC-MSCs Alleviate Renal Fibrosis in DN Models via Blocking Myofibroblast Transdifferentiation (MFT) Mediated by TGF-*β*1/Smad2/3 Signaling Pathway

**Figure 3:**
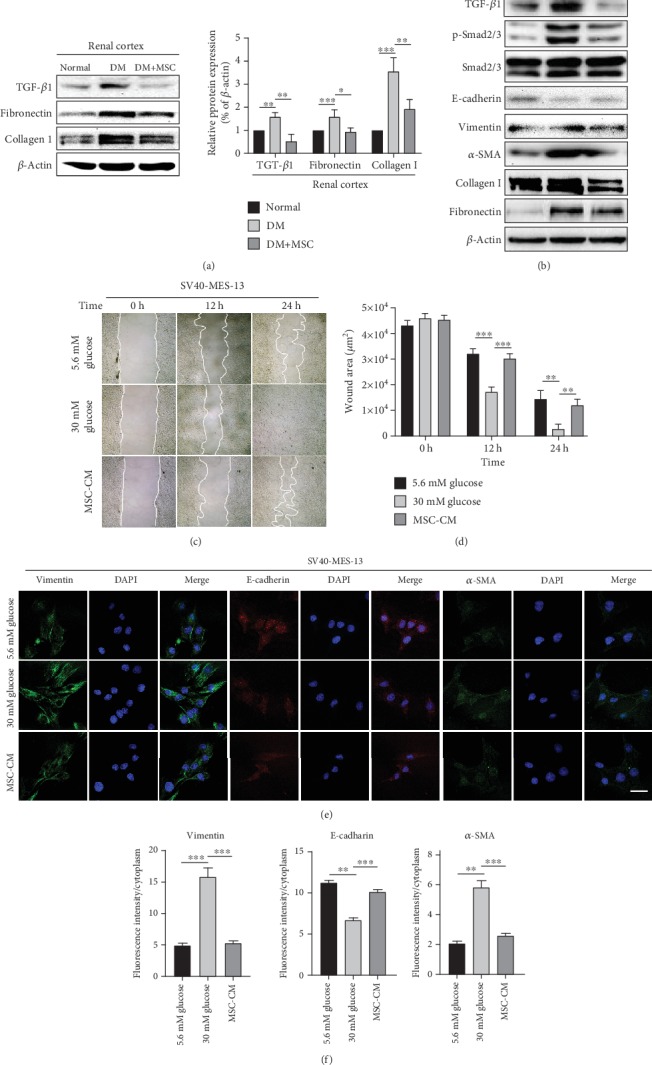
mUC-MSC paracrine attenuates renal fibrosis by blocking myofibroblast transdifferentiation triggered by the TGF-*β*1/Smad2/3 signaling pathway in the DN cell model. (a) Western blotting analysis determines the inhibitory effect of mUC-MSC transplantation on TGF-*β*1, fibronectin, and collagen I levels in renal cortexes of mice in normal, diabetes mellitus (DM), and DM+MSC groups. Data are expressed as mean ± SD of at least 4 animals. ^∗^*P* < 0.01, ^∗∗^*P* < 0.01, and ^∗∗∗^*P* < 0.001. (b) Immunoblotting analysis of TGF-*β*1, total and phosphorylated Smad2/3, E-cadherin, vimentin, *α*-SMA, fibronectin, and collagen I in mesangial cells treated with low glucose, high glucose, and mUC-MSC coculture. The western blotting experiment was repeated independently three times. (c) Wound healing assay determines the effect of mUC-MSC-conditioned medium on cell migration. (d) The wound areas in the cultured mesangial cells were quantified. Data are expressed as mean ± SD. ^∗∗^*P* < 0.01 and ^∗∗∗^*P* < 0.001. (e) Immunofluorescence assay determines the effect of mUC-MSC-conditioned medium on E-cadherin, vimentin, and *α*-SMA. Bar: 100 *μ*m. (f) The intensity of the positive areas in mesangial cells is quantified. Data are expressed as mean ± SD. ^∗∗^*P* < 0.01 and ^∗∗∗^*P* < 0.001.

To investigate the therapeutic effects of mUC-MSCs on renal fibrosis *in vivo*, western blotting analysis was performed to detect fibrotic factors such as TGF-*β*1, fibronectin, and collagen I in kidney samples from renal cortexes of mice in normal, diabetes mellitus (DM), and DM+MSC groups. Kidney tissues from DM mice showed significantly increased deposition of both TGF-*β*1 and ECM proteins compared with normal mice. However, the levels of TGF-*β*1, fibronectin, and collagen I in the kidney tissues of DM+MSC mice were significantly reduced compared with DM mice (Figure 3(a)).

In response to injury, mesangial cells can transdifferentiate into myofibroblasts that secrete ECM proteins, which is an important pathological basis for renal fibrosis [7, 24, 25]. The central role of TGF-*β*1 in MFT and pathogenesis of renal fibrosis has been generally accepted [26, 27]. To identify the molecular basis of the antifibrosis effect observed after mUC-MSC administration, we established a DN cell model based on high-glucose- (HG-, 30 mM) cultured mesangial cells. The higher levels of ECM proteins and increased cell proliferation ability were shown in the DN cell model compared to the mesangial cells cultured in normal glucose (NG, 5.6 mM) (Figure S1). We found that after coculturing with mUC-MSCs, the protein expression of fibronectin and collagen I was reduced compared to HG-cultured mesangial cells. Moreover, mUC-MSC coculture decreased the levels of TGF-*β*1, phosphorylated Smad2/3 (p-Smad2/3), and mesenchymal marker vimentin and fibroblast marker *α*-SMA, and increased epithelial marker E-cadherin level compared to HG-cultured mesangial cells (Figure 3(b)). We furthered studied the effects of the mUC-MSC-conditioned medium (MSC-CM) on mesangial cell migration using a wound healing assay. As shown in Figures 3(c) and 3(d), the rate of wound closure significantly increased with time in mesangial cells cultured in HG compared to NG. By contrast, MSC-CM markedly decreased cell migration induced by HG. In addition, we used cell immunofluorescence staining to detect the expression of the three MFT markers. The results showed that MSC-CM significantly reduced the fluorescence intensities of vimentin and *α*-SMA, and increased the E-cadherin fluorescence intensity in mesangial cells induced by HG (Figures 3(e) and 3(f)).

### 3.4. The mUC-MSC-CM Inhibits Cell Proliferation and Elevates the Levels of MMPs in HG-Treated Mesangial Cells

**Figure 4:**
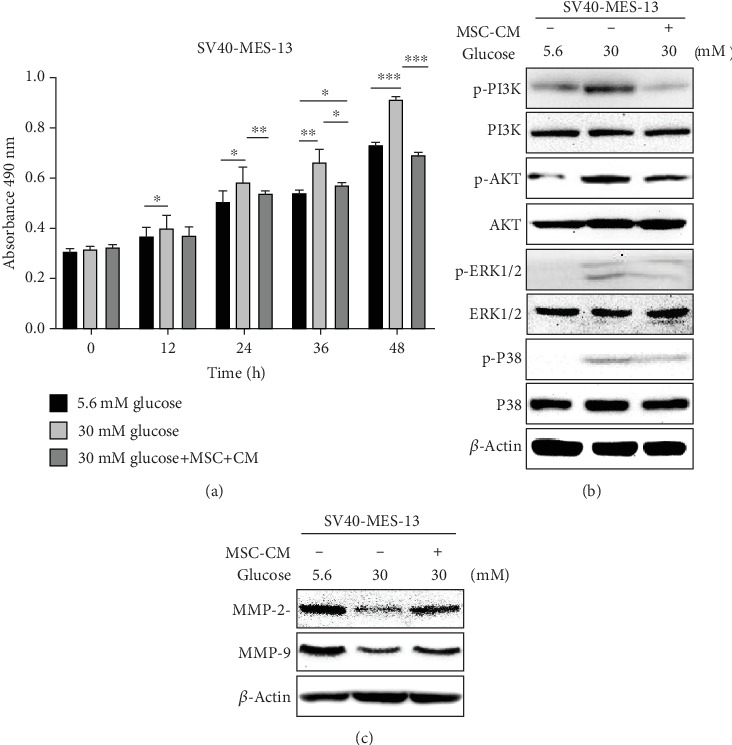
mUC-MSC paracrine inhibits cell proliferation by blocking PI3K/Akt and MAPK signaling pathways and promotes expression of MMPs in the DN cell model. (a) The inhibitory effect of mUC-MSC-conditioned medium on mesangial cell proliferation. Data are expressed as mean ± SD. ^∗^*P* < 0.05, ^∗∗^*P* < 0.01, and ^∗∗∗^*P* < 0.001. (b) Immunoblotting analysis determined the regulatory effects of mUC-MSC-conditioned medium on the phosphorylation of PI3K, Akt, P38, and ERK1/2. (c) mUC-MSC-conditioned medium upregulates the levels of MMP2 and MMP9. All of the western blotting experiments were repeated independently three times.

Previous studies have suggested that the abnormal proliferation of mesangial cells was responsible for the observed accumulation of ECM and thus played an essential role in diabetic nephropathy [24, 28]. Therefore, we examined the modulating effect of mUC-MSCs on mesangial cell proliferation and the PI3K/Akt and MAPK signaling pathways. An MTS assay showed that cell proliferation was enhanced in mesangial cells cultured in HG compared to those in NG with time, whereas MSC-CM exhibited a significantly suppressive effect on mesangial cell proliferation induced by HG (Figure 4(a)). Moreover, western blotting analysis suggested that the levels of p-PI3K, p-Akt, p-ERK1/2, and p-P38 were higher in mesangial cells cultured in HG than in NG, while mUC-MSC-CM effectively inhibited the phosphorylation of these protein kinases (Figure 4(b)). ECM accumulation, as a hallmark morphologic finding of DN, is not only related to the excessive synthesis of ECM proteins, but also to their decreased degradation by the MMPs. It is known that MMPs play a role in renal fibrosis in DN [29]. We therefore studied the effect of mUC-MSC paracrine on the expression of MMPs. Western blotting analysis showed that MSC-CM could abolish the inhibition of HG for the expression of MMP2 and MMP9 in mesangial cells (Figure 4(c)).

### 3.5. Inhibition of Exosome Shed by mUC-MSCs Abolishes the Antifibrotic Effect of mUC-MSC Paracrine in HG-Cultured Mesangial Cells

In order to evaluate the role of exosomes in the antifibrosis effect of MSC paracrine in DN, mUC-MSCs were administrated by 10 *μ*M GW4869, an inhibitor of exosomes, for 24 h to collect MSC-CM lacking exosomes that was named MSC (GW4869)-CM. MSC-CM collected from mUC-MSCs administrated by DMSO was considered as a control and named MSC (DMSO)-CM. Firstly, we demonstrated that exosomes isolated from MSC-CM were positive for exosomal markers such as CD9, CD63, HSP70, and TSG101 (Figure S2A), and that the diameter of exosomes shed by mUC-MSCs was about 164 nm, which is consistent with the standard for the range of exosomes with diameters of about 30-200 nm. However, the exosomes derived from MSC (GW4869)-CM could not be detected under the same condition (Figure S2B). A BCA assay further showed that the protein concentration of exosomes derived from MSC (GW4869)-CM was about only one-fourth of the exosomes from MSC (DMSO)-CM (Figure S2C). Additionally, the MTS assay suggested that the cell growth of mUC-MSCs treated with 10 *μ*M GW4869 was not affected compared to the cells treated with DMSO under the same condition (Figure S2D). Taken together, these results indicated that GW4869 dramatically inhibited the secretion of exosomes shed by mUC-MSCs without affecting the cell growth of the MSCs.

**Figure 5:**
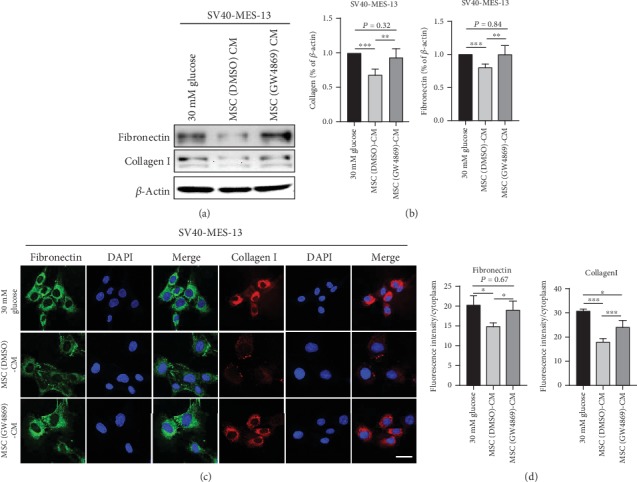
mUC-MSC-conditioned medium lacking exosomes abolishes the inhibitory effects of mUC-MSC paracrine on the expression of fibronectin and collagen I. (a) Immunoblotting analysis of fibronectin and collagen I in mesangial cells treated with high glucose, mUC-MSC-conditioned medium, or mUC-MSC-conditioned medium lacking exosomes. The western blotting experiment was repeated independently three times. (b) Relative amounts of protein are normalized to an internal control, *β*-actin. Data are expressed as mean ± SD. ^∗^*P* < 0.05, ^∗∗^*P* < 0.01, and ^∗∗∗^*P* < 0.001. (c) Immunofluorescence assay determines the effect of mUC-MSC-conditioned medium lacking exosomes on the levels of fibronectin and collagen I. Bar: 100 *μ*m. (d) The intensity of the positive areas in mesangial cells is quantified. Data are expressed as mean ± SD. ^∗^*P* < 0.05, ^∗∗^*P* < 0.01, and ^∗∗∗^*P* < 0.001.

Next, we performed western blotting and an IF experiment to investigate how the absence of exosomes affects the antifibrosis effect of mUC-MSC paracrine in DN. We found that MSC (GW4869)-CM abolished the inhibitory effect of MSC (DMSO)-CM on the expression of fibronectin and collagen I in HG-cultured mesangial cells (Figures 5(a) and 5(b)). Also, the IF experiment revealed that MSC (GW4869)-CM also nearly counteracted the suppressive effects of MSC (DMSO)-CM on fluorescence intensities of the both ECM proteins (Figures 5(c) and 5(d), ^∗^*P* < 0.05, ^∗∗^*P* < 0.01, and ^∗∗∗^*P* < 0.001).

### 3.6. Inhibition of Exosomes Shed by mUC-MSCs Abolishes the Antifibrotic Effects of mUC-MSC Paracrine by Recovering the Activity of TGF-*β*1/Smad2/3 Signaling and Mesangial Cell Proliferation, and Decreasing the Levels of MMPs

**Figure 6:**
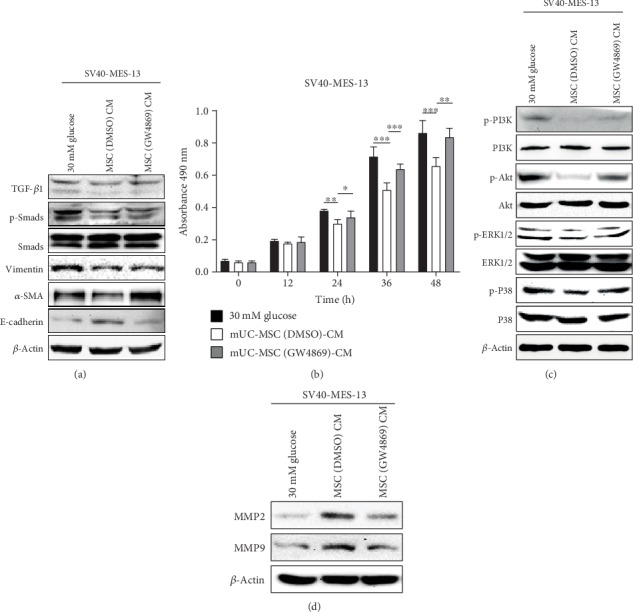
mUC-MSC-conditioned medium lacking exosomes impairs the effects of mUC-MSC paracrine on cell proliferation and expression of MMPs in high glucose-cultured mesangial cells. (a) Immunoblotting analysis of TGF-*β*1, total and phosphorylated Smad2/3, E-cadherin, vimentin, and *α*-SMA in mesangial cells treated with high glucose, mUC-MSC-conditioned medium, or mUC-MSC-conditioned medium lacking exosomes. (b) MTS assay was used to determine the effect of mUC-MSC-conditioned medium lacking exosomes on mesangial cell proliferation. (c) Immunoblotting analysis for the phosphorylation of PI3K, Akt, P38, and ERK1/2 in mesangial cells (d) Immunoblotting analysis for the levels of MMP2 and MMP9 in mesangial cells. All of the western blotting experiments were repeated independently three times.

We further attempted to reveal the mechanisms by which the absence of exosomes impairs the antifibrosis effect of mUC-MSC paracrine in DN. Studies by western blotting analysis confirmed that the lack of exosomes also abolished the regulatory effects of MSC-CM on TGF-*β*1, p-Smad2/3, vimentin, *α*-SMA, E-cadherin, MMP2, and MMP9 in HG-cultured mesangial cells (Figures 6(a) and 6(d)). Moreover, the inhibition of mUC-MSC paracrine for mesangial cell proliferation and the levels of p-PI3K, p-Akt, p-ERK1/2, and p-P38 were also reduced after administrated by MSC (GW4869)-CM (Figures 6(b) and 6(c)). These results indicated that exosomes play a key role in the antifibrosis effects of mUC-MSC paracrine in DN by inhibiting MFT triggered by the TGF-*β*1/Smad2/3 signaling pathway and mesangial cell proliferation mediated by the PI3K/Akt and MAPK signaling pathways, and enhancing the expression of MMPs.

## 4. Discussion

The present study demonstrates that mUC-MSCs improve urine microalbumin and pathologic morphological features such as glomerular hypertrophy, base membrane thickening, podocyte process effacement, and fibrotic alteration in kidneys of STZ-induced diabetic mice. In the DN cell model, the mUC-MSC paracrine alleviates renal fibrosis by reducing accumulation of ECM proteins through different mechanisms. The mechanisms by which mUC-MSC paracrine attenuates renal fibrosis involve the inhibition of MFT triggered by the TGF-*β*1/Smad2/3 signaling pathway and mesangial cell proliferation mediated by the PI3K/Akt and MAPK signal transduction pathways, and elevating MMP levels. Importantly, we provide the evidence that exosomes play a central role in the antifibrosis effect of mUC-MSC paracrine in DN through multiple mechanisms such as the modulation of MFT, mesangial cell proliferation, and MMP expression.

As a major cause of the pathogenesis of DN, renal fibrosis is characterized by the activation and proliferation of fibroblasts and the deposition of ECM [22]. Three intrinsic cell types in the glomerulus, podocytes, and endothelial and mesangial cells contribute to renal fibrosis in DN. Although renal fibrosis in diabetes is generally viewed as irreversible, some studies demonstrate that remission or regression is possible. In this study, western blotting analysis showed that infusion with mUC-MSCs alleviated renal fibrosis in renal cortexes of diabetic mice (Figures 2(b) and 3(a)), which agrees with previous studies about other tissue resource-derived MSCs on kidney fibrosis [30, 31]. Recently, some *in vitro* studies indicate that UC-MSCs have an important role in the repair of renal cell injuries in DN by a paracrine mechanism [20, 22]. However, the mechanisms underlying the antifibrotic modulation by MSCs are still not well understood. Therefore, we firstly established a DN cell model of DN based on HG-cultured mesangial cells (Figure S1) to confirm the antifibrosis role of mUC-MSC paracrine in DN. Our results showed that mUC-MSC coculture or CM downregulated the expression of fibronectin and collagen I (Figure 3(b)).

Experimental and clinical studies have shown that hyperglycemia and many other related factors induce profibrotic changes in kidney intrinsic cells by promoting the processes of MFT and EMT [7]. Myofibroblasts play a critical role in inducing renal fibrosis in the glomerulus and tubulointerstitium [32]. Emerging evidence also indicates that MFT is a major source of the myofibroblasts in DN [25, 33, 34]. In response to hyperglycemic stimuli, mesangial cells can acquire a myofibroblast-like phenotype characterized by the loss of normal cell-cell and cell-matrix junctions, while gaining the expression of new mesenchymal markers, such as vimentin, *α*-smooth muscle actin (*α*-SMA), collagen I/IV, and fibronectin; ECM proteases; PAI-1; and TIMPs [7]. The profibrotic cytokine TGF-*β*1 regulates not only MFT induction but also the synthesis of ECM molecules, including collagen I, fibronectin, and laminin [3, 35–38]. We therefore hypothesized that mUC-MSC paracrine was likely to play a role in attenuating renal fibrosis in DN by inhibiting TGF-*β*1-triggered transdifferentiation of mesangial cells into a myofibroblast-like phenotype. Our data showed that MSC-CM inhibited cell migration, downregulated the expression of fibroblast markers such as vimentin and *α*-SMA, and increased the expression of epithelial marker E-cadherin in mesangial cells cultured in HG (Figure 3). Moreover, MSC-CM decreased the levels of TGF-*β*1 and p-Smad2/3 in mesangial cells undergoing an MFT-like process induced by HG (Figure 3(b)), indicating the role of mUC-MSC paracrine in blocking the TGF-*β*1/Smad2/3 signaling pathway. These findings indicated that mUC-MSC paracrine could inhibit TGF-*β*1-caused MFT and subsequent renal fibrosis in the progression of DN.

In diabetic animals, pathological examination shows significant glomerular mesangial expansion, matrix expansion accumulation, and basement membrane thickening [39–41]. Previous studies have shown that mesangial cell proliferation is responsible for observed ECM accumulation and mesangial expansion [42, 43]. Recent findings suggest that TGF-*β*1 downstream of Smad-independent pathways such as PI3K/Akt [11, 44, 45] and MAPKs [10, 46] that contribute to cell proliferation, are involved in the occurrence and development of renal fibrosis. Thus, controlling mesangial cell proliferation and thus reducing ECM protein accumulation may be considered an effective strategy to prevent and retard renal fibrosis in DN. Based on the previous observations, we further suggested that mUC-MSC paracrine significantly inhibited mesangial cell proliferation and activation of the PI3K/Akt and MAPK signaling pathways (Figures 4(a) and 4(b)). In addition to causing EMT, EndoMT, and MFT, TGF-*β*1 also promotes the deposition of ECM proteins by directly inhibiting the activity of MMPs and eliciting glomerular basement membrane thickening and mesangial expansion [47–50]. Herein, we investigated the role of mUC-MSC paracrine in regulating the expression of MMPs, and found that MSC-CM could elevate the levels of MMP2 and MMP9 in mesangial cells cultured in HG (Figure 4(c)), which is in accordance with a recent study showing that the upregulation of MMP9 is related to the attenuation of bone marrow-derived MSCs for DN in diabetic rats [19]. The results indicated that mUC-MSC paracrine inhibited excessive deposition of ECM proteins by reducing the number of mesangial cells transdifferentiating into myofibroblasts and increasing the expression of MMPs, thereby ameliorating renal fibrosis in DN.

Emerging evidence has shown the key roles of paracrine mechanism in ameliorating DN by MSCs [22, 30, 40, 51]. During this process, MSCs can decrease renal fibrosis, suppress oxidative stress, inhibit renal cell injuries, and attenuate adverse inflammatory events and immune response through extracellular vesicles (EVs) [8, 30, 31, 40, 51] and secreted soluble factors [52–55]. For EVs, the contribution of exosomes to MSC therapeutic efficacy has been widely indicated in many different studies [31, 51]. However, it is still unclear to what extent do either of the exosomes or secreted soluble factors affect MSC paracrine activity in ameliorating renal fibrosis. Interestingly, our findings demonstrated that the strong intervention of exosomes shed by mUC-MSCs abolished the inhibitory effects of MSC-CM on the expression of ECM proteins in mesangial cells cultured in HG (Figure 5). In terms of the mechanism, our results confirmed that the absence of exosomes eliminated the antifibrosis effects of mUC-MSC paracrine in DN by promoting TGF-*β*1-triggered MFT, enhancing mesangial cell proliferation mediated by the PI3K/Akt and MAPK signaling pathways, and downregulating the expression of MMPs (Figure 6). Based on the above results, mUC-MSC paracrine might play an important role in alleviating renal fibrosis in DN mainly through exosomes in combination with the least amount of secreted soluble factors.

In conclusion, our study demonstrates that the underlying mechanisms by which mUC-MSC paracrine attenuates renal fibrosis in DN involve blocking TGF-*β*1-triggered MFT, inhibiting mesangial cell proliferation mediated by the PI3K/Akt and MAPK signaling pathways, and elevating the levels of MMPs in mesangial cells. Notably, we provide the evidence that the antifibrosis role of mUC-MSC paracrine in DN might be mainly due to exosomes. Based on these findings, advances in MSC manufacturing technologies perhaps make a large contribution to the generation of widespread, successful, clinical MSC therapies, and exosomes shed by UC-MSCs may be considered as a novel cell-free therapeutic approach for renal fibrosis in patients with DN.

## Figures and Tables

**Table 1:** mUC-MSC administration improved diabetic nephropathy in STZ-induced diabetic mice.

	Before MSC administration		8 weeks after MSC administration		
	Normal (*n* = 6)	DM (*n* = 8)	Normal (*n* = 6)	DM (*n* = 8)	DM+MSC (*n* = 8)
Kidney/body weight (mg/g)	8.92 ± 0.31	18.45 ± 1.82^∗^	9.48 ± 0.43	20.0 ± 1.35^∗^	16.84 ± 0.93^∗^
Blood glucose (*μ*M)	7.35 ± 1.34	26.65 ± 3.70^∗^	6.68 ± 0.55	24.53 ± 1.03^∗^	14.36 ± 3.80^∗^^,#^
Urine creatinine (*μ*M)	2345.1 ± 364.1	451.6 ± 115.3^∗^	2147.8 ± 451.6	157.6 ± 14.5^∗^	235.4 ± 56.9^∗^^,#^
24-hour urine microalbumin (mg)	3.29 ± 1.28	25.60 ± 7.23^∗^	3.99 ± 1.50	64.57 ± 27.33^∗^	44.45 ± 20.68^∗^^,#^

Physical and biochemical parameters of experimental animals. Before mUC-MSC administration (8 weeks after diabetic mellitus induced by STZ), mice received 200 *μ*L of the vehicle (DM) or 1 × 10^4^ mUC-MSCs/g weight resuspended in 200 *μ*L of vehicle (DM+MSC) via the tail vein. All biochemical parameters were evaluated in urine and blood samples obtained after 4 hours of fasting. Data are presented as mean ± SD of 6~8 animals. ^∗^*P* < 0.05 versus normal; ^#^*P* < 0.05. versus DM for the same time point.

## Data Availability

The data that support the findings of this study are available from the corresponding authors upon reasonable request.

## Supplementary Materials

Supplementary materials Figure S1: the DN cell model based on high-glucose-cultured mesangial cells. (A) Measurement of cell proliferation in high-glucose-induced SV40-MES-13 cells. (B) Western blot analysis of the levels of ECM proteins in high-glucose-induced SV40-MES-13 cells. Figure S2: inhibitory effect of GW4869 on the secretion of exosomes from mUC-MSCs. (A) Identification of exosome markers. (B) Detection of exosome particle size and purity. (C) GW4869 decreases the levels of exosome protein from mUC-MSCs. Each value represents the mean (±SD) of a representative of 3 independent experiments. Statistical significance: ^∗^*P* < 0.05 versus negative control. (D) 10 *μ*M GW4869 is shown not to affect cell proliferation of mUC-MSC cells.
