# Supplementary material for: Mouse Umbilical Cord Mesenchymal Stem Cell Paracrine Alleviates Renal Fibrosis in Diabetic Nephropathy by Reducing Myofibroblast Transdifferentiation and Cell Proliferation and Upregulating MMPs in Mesangial Cells
Source: J Diabetes Res. 2020 May 2;2020:3847171. doi: 10.1155/2020/3847171 (PMC7222483; doi:10.1155/2020/3847171)
Supplement: Supplementary materials — Figure S1: the DN cell model based on high-glucose-cultured mesangial cells. (A) Measurement of cell proliferation in high-glucose-induced SV40-MES-13 cells. (B) Western blot analysis of the levels of ECM proteins in high-glucose-induced SV40-MES-13 cells. Figure S2: inhibitory effect of GW4869 on the secretion of exosomes from mUC-MSCs. (A) Identification of exosome markers. (B) Detection of exosome particle size and purity. (C) GW4869 decreases the levels of exosome protein from mUC-MSCs. Each value represents the mean (±SD) of a representative of 3 independent experiments. Statistical significance: ∗P < 0.05 versus negative control. (D) 10 μM GW4869 is shown not to affect cell proliferation of mUC-MSC cells. [file 3847171.f1.pdf]

Mouse umbilical cord mesenchymal stem cell paracrine alleviates renal fibrosis in diabetic nephropathy by reducing myofibroblast transdifferentiation and cell proliferation and upregulating MMPs in mesangial cells

Hongde Li [a,b, c], Pengfei Rong [a,b, c], Xiaoqian Ma [a,b], Wei Nie [a,b], Yan chen[d], Juan Zhang [a,b], Qiong Dong [a,b], Min Yang [a,b], Wei Wang [a,b, c, d] *

[a] Cell Transplantation and Gene Therapy Institute, The Third Xiangya Hospital of Central South University, Changsha, Hunan, China

[b] Department of Radiology, The Third Xiangya Hospital of Central South University, Changsha, Hunan, China

[c] Postdoctoral Research Station of Special Medicine, The Third Xiangya Hospital of Central South University, Changsha, Hunan, China

d Department of pathology, the third Xiangya Hospital of Central South University, Changsha, Hunan, China

e Engineering and Technology Research Center for Xenotransplantation of Hunan Province, Changsha, China

* Corresponding author: Cell Transplantation and Gene Therapy Institute, The Third Xiangya Hospital of Central South University, Changsha, Hunan, China. E-mail address: cjr.wangwei@vip.163.com

Supplementary Figures and Legends

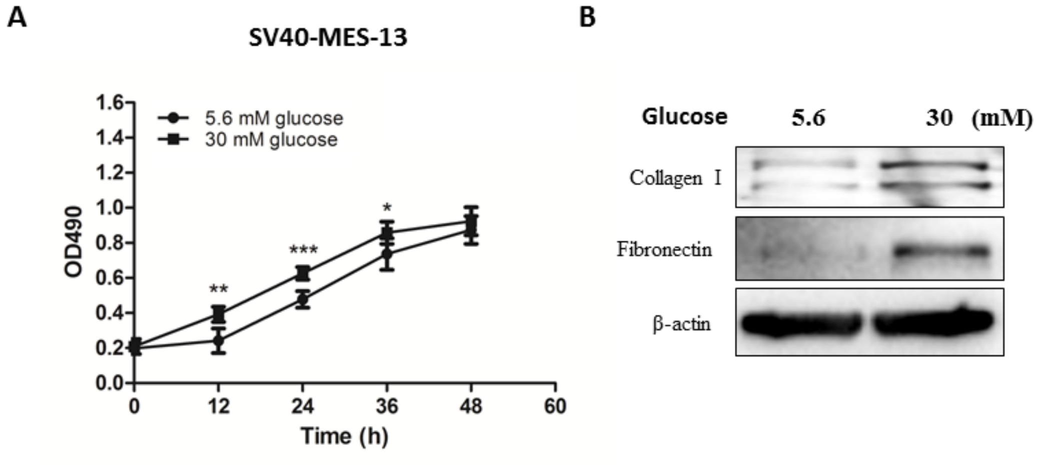

Figure S1. The DN cell model based on high glucose-cultured mesangial cells. (A)

Measurement of cell proliferation in high glucose-induced SV40-MES-13 cells. **(B)** Western blot analysis of the levels of ECM proteins in high glucose-induced SV40-MES-13 cells.

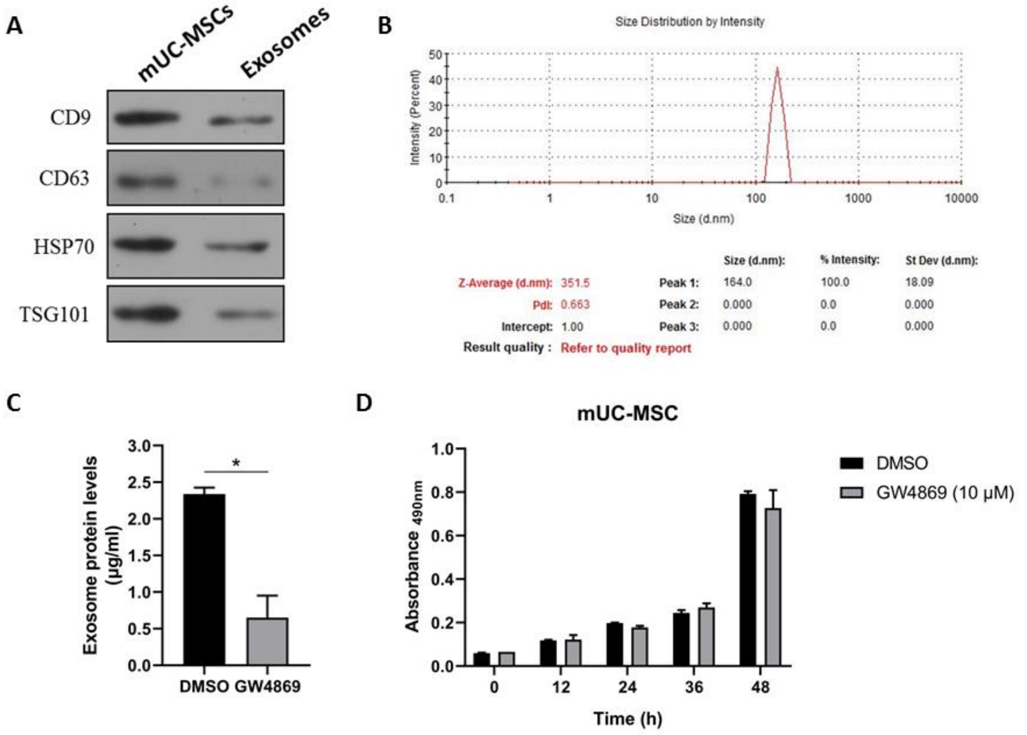

Figure S2. Inhibitory effect of GW4869 on the secretion of exosomes from mUC-MSCs (A) Identification of exosome markers. **(B)** Detection of exosome particle size and purity. **(C)** GW4869 decreases the levels of exosome protein from mUC-MSCs. Each value represents the mean (± SD) of a representative of 3 independent experiments. Statistical significance:*p < 0.05 versus negative control. **(D)** 10 μM GW4869 is shown not to affect cell proliferation of mUC-MSC cells.
